# Shaping Diversity Into the Brain’s Form and Function

**DOI:** 10.3389/fncir.2018.00083

**Published:** 2018-10-10

**Authors:** Lauren N. Miterko, Elizabeth P. Lackey, Detlef H. Heck, Roy V. Sillitoe

**Affiliations:** ^1^Department of Pathology and Immunology, Baylor College of Medicine, Houston, TX, United States; ^2^Program in Developmental Biology, Baylor College of Medicine, Houston, TX, United States; ^3^Jan and Dan Duncan Neurological Research Institute of Texas Children’s Hospital, Houston, TX, United States; ^4^Department of Neuroscience, Baylor College of Medicine, Houston, TX, United States; ^5^Department of Anatomy and Neurobiology, University of Tennessee Health Science Center, Memphis, TN, United States

**Keywords:** neuron, glia, folding, layering, connectivity, topography, patterning

## Abstract

The brain contains a large diversity of unique cell types that use specific genetic programs to control development and instruct the intricate wiring of sensory, motor, and cognitive brain regions. In addition to their cellular diversity and specialized connectivity maps, each region’s dedicated function is also expressed in their characteristic gross external morphologies. The folds on the surface of the cerebral cortex and cerebellum are classic examples. But, to what extent does structure relate to function and at what spatial scale? We discuss the mechanisms that sculpt functional brain maps and external morphologies. We also contrast the cryptic structural defects in conditions such as autism spectrum disorders to the overt microcephaly after Zika infections, taking into consideration that both diseases disrupt proper cognitive development. The data indicate that dynamic processes shape all brain areas to fit into jigsaw-like patterns. The patterns in each region reflect circuit connectivity, which ultimately supports local signal processing and accomplishes multi-areal integration of information processing to optimize brain functions.

## Introduction

The brain is responsible for a seemingly endless number of behaviors that require cognition, sensation, and action. How the brain processes information from its environment, develops thoughts, weighs emotions, and drives the repertoire of responsive behaviors depends on a precisely coordinated interaction of different structures, each with its own specialized functions. The cerebral cortex, subcortical structures, and the cerebellum broadly plan behaviors by controlling emotions, organizing and interpreting external and internal sensory information, forming memories, maintaining homeostasis, coordinating appropriate muscle activity, and instructing language. Brain functions are clearly diverse; therefore, it makes sense that none of the brain regions have morphologies that look alike from the outside and that their networks are comprised of distinct layers and cell types. However, there are many genetic, cellular, molecular, and developmental processes that are shared between brain regions. Given that these similarities are superimposed upon a multitude of differences, can structure tell us anything about function?

While the answers to these questions might, at first glance, seem to be an overwhelming “yes,” there is not always clear linkage between structure and function for many brain areas. To address this problem, one must consider the developmental mechanisms that generate the brain. The central nervous system arises from a simple neuroepitehlium that is initially unremarkable in its specificity along its rostral-caudal axis. Gene function during embryogenesis transforms the neuroepithelium into distinct domains that will form particular brain regions. It is at this stage of development that one may ask how structures are uniquely shaped to acquire their final function. Cells in each region begin to proliferate according to a specific spatial and temporal timetable. At least two classes are produced: neurons and glia. The glia serve as a lineage source for neurons, but they also form the cellular substrate for neuronal migration to occur. Therefore, depending on the classes of neurons and glia and their organization, it has been argued that the suggested ratio of 90% glia to 10% neurons in the mammalian brain could provide clues as to how the brain acquires its intricate morphologies. But, this ratio has been challenged recently, suggesting a ratio closer to 50:50 (Herculano-Houzel, 2014). Moreover, although relatively simple in form, the *drosophila* *melanogaster* brain only has 10% glia (Kremer et al., 2017), yet there is still considerable complexity in the fly brain that includes intricate folds and specialized functions. But, why fold in the first place? It may be argued that massive regions such as the cerebral cortex must fold in order to accommodate the sheer quantity of critical circuits. However, the cerebellum contains fewer types of circuits (certainly at the basic anatomical level, although this could be challenged based on molecular complexity, as discussed later), yet it has more neurons than any other part of the brain. Based on these problems, we then face the question, what drives the folding, and are there equivalent mechanisms in different parts of the brain? In this review, we address these issues and take into account that each brain region contains an array of distinct cell types with unique morphologies, densities, and functions, and we also consider how neurons migrate and how their axons are guided into precise locations to form brain networks. We ask, what physical forces assemble these network components into a brain region (Garcia et al., 2018)? We discuss how functionality is assembled across brain regions and how neural circuits link up into previously unappreciated wiring diagrams that are critical for behavior. Our attempt is not to solve every question and conundrum in the field of cerebellar and cerebral cortical folding. Instead, our efforts are to take a wholistic view of how the brain is packaged from the outside to its inside, and to stimulate a discussion about how one level of complexity, be it cellular or molecular, feeds into the next, in developing and adult circuits. This view might also teach us about brain function.

## Main Text

### Multiple Levels of Heterogeneity in the Brain

#### Regional Variation and Specificity

Historically, the cerebral cortex and cerebellum have been extensively studied for their structures and functions. For example, Brodmann (1909) identified 43 areas in the human brain based on the general cytoarchitecture of the cerebral cortex. After taking into account regional cellular composition and density, Penfield (1968) considered the functional contributions of the cerebral cortex, elucidating the areas responsible for processing somatosensory, visual, auditory, and motor information through producing an exhaustive functional map based on responses to stimulation techniques that he pioneered for therapy in epilepsy. Thereafter, impairments to these cortical subdivisions have been linked to a large number of diverse developmental and pathological features of brain disease.

Cerebellar studies also have a rich and fascinating history (Manto, 2008), with suggestions of functional topography dating back to the early 1900s (Bolk, 1906). The cerebellar cortex, which is often described as having a uniform cellular composition, is in fact heterogeneous in its molecular properties. As we will discuss later, the cerebellar cortex is divided into an array of parasagittal patterns that segment all of its cell classes. Importantly, however, the molecular patterning is accompanied by anatomical divisions, both in the cerebellar cortex (Ozol and Hawkes, 1997) and the white matter (Voogd and Ruigrok, 1997). Indeed, much like the cerebral cortex, the different domains within the cerebellum reflect developmental (Sillitoe and Joyner, 2007; Apps and Hawkes, 2009; Apps et al., 2018), functional (Cerminara et al., 2015), and pathological features (Sarna and Hawkes, 2003). Strikingly, each of these medial-lateral patterning properties is superimposed upon a broader anatomical plan that is also segmented upon the same axis. From medial to lateral the vermis, paravermis, and hemispheres occupy distinct locations, have specific folding architectures, contain specific circuits, and contribute to largely different behaviors.

Findings in the cerebral cortex and cerebellum have been complemented by studies into understanding the unique structures and functions characteristic to other areas such as the hippocampus. We will not review the thousands of studies devoted to hippocampal functional specificity, but suffice it to say that a large body of work has revealed an intricate and remarkable segmentation of its functions (Hitti and Siegelbaum, 2014; Kohara et al., 2014; Okuyama et al., 2016). Support for these modern animal model studies, like many sectors of neuroscience, came from older studies from human patients and the field of psychiatry. The concept that the cerebral cortex segments and shuttles information into distinct brain regions and that this process can be compromised in disease was found to be shared with the hippocampus. For example, the resulting amnesia after lesioning the hippocampus revealed its critical roles in memory formation (Scoville and Milner, 1957). The main issue to consider, as a starting point, is that each unique brain structure appears to come equipped with a unique set of capabilities. The question we ask is how do these capabilities arise, and are there common mechanistic themes for how they arise?

#### Cell Layering and Connectivity

One common feature between the cerebral cortex, cerebellum, and hippocampus is that they all have an exquisite layering of cells. Importantly, the layering in all three structures is disrupted in mice that lack REELIN and related proteins (Howell et al., 1997). These data indicate a common requirement for specific genetic programs and at least some shared dependence on developmental processes such as neuronal migration (Wasser and Herz, 2017). Still, each structure must use these “common” cues to assemble unique circuits. The layering and interactions of cells between the layers of the cerebellar cortex are a prime example. To appreciate these ideas, it is useful to recall that connectivity within the cerebellum is understood at a considerable level of detail, with each cell type forming stereotypical connections with its neighbors. The cerebellum has three distinct layers, and for comparison, the much more complicated cerebral cortex has six main layers. The most superficial cerebellar layer contains inhibitory interneurons and excitatory parallel and climbing fibers. Both project onto Purkinje cells, which make up the middle layer called the Purkinje cell layer. The Purkinje cells perform the main computations in the cerebellum. The deepest layer is called the granular layer and it contains millions of excitatory neurons called granule cells as well as mossy fibers that deliver sensory signals to the Purkinje cells. Below the three layers is a dense network of fiber tracks. Embedded in this network are the cerebellar nuclei. The cerebellar nuclei are specialized neurons that transmit the final output of the cerebellum. They link the cerebellum to the rest of the brain and spinal cord. Early studies of the cerebellum revealed an incredible level of structural and functional variation in the circuitry as was found in the cortex (Fox and Snider, 1967). Work from Marr (1969) and Albus (1971) used this structural map of the cerebellum, and at that time the quickly emerging details of its functional connectivity (Eccles, 1965), to postulate theories on its computational power over motor control. Given that the cerebellum has one neuronal population responsible for the output of its cortex, the Purkinje cell, and that this cell type is innervated by inputs in a predictable, reproducible pattern, the cerebellum is thought to execute a multitude of motor behaviors by modulating Purkinje cell spiking and the downstream consequences on cerebellar nuclear neuron firing (Marr, 1969; Gilbert, 1974; Ruigrok, 2011). It is also important to note that Purkinje cells project to distinct cerebellar output nuclei as revealed by the pattern of axonal projections, compartmental expression of molecules, and functional designation. These combined features further subdivide the cerebellum and potentially add complexity to its computational capabilities (Ruigrok, 2011; Apps et al., 2018; Miterko et al., 2018). In a similar manner, but for different behavioral consequences, the hippocampus is separated into distinct areas [cornu ammonis (CA) fields, dentate gyrus, and subiculum] and layers that are conceptually reminiscent of those in the cerebellum, where cells are organized in a predictable, spatial pattern (Arbib et al., 1998). This particular organization is thought to promote information processing and neuronal coupling (Arbib et al., 1998) to support different non-motor and motor behaviors.

#### Functional Specializations

The heterogeneity of the brain, as exemplified by these three examples—the cerebral cortex, cerebellum, and hippocampus—spans a great number of structures and their associated functions. Among the anatomical differences, there are also physiological and chemical differences that affect circuit formation and function. Nonetheless, what each of these three brain regions have are subdivisions, which is theorized to support their functions. Comparative analyses upheld this belief, adding to it that as mammals evolved, so did brain structure to accommodate higher order functions. This manifested in a trend in toward a greater subdivision of the cerebral cortex into functionally distinct areas, where early mammals likely had on the order of 20 distinct cortical areas while humans may have more than 200 distinct cortical areas (Kaas, 2013). Neuroimaging of the human cerebellar cortex also reveals functional topography spanning not only motor control but also specific higher order limbic and cognitive tasks, including lateralization of language-related activity (Stoodley and Schmahmann, 2009).

#### Modules and Maps

**FIGURE 1:**
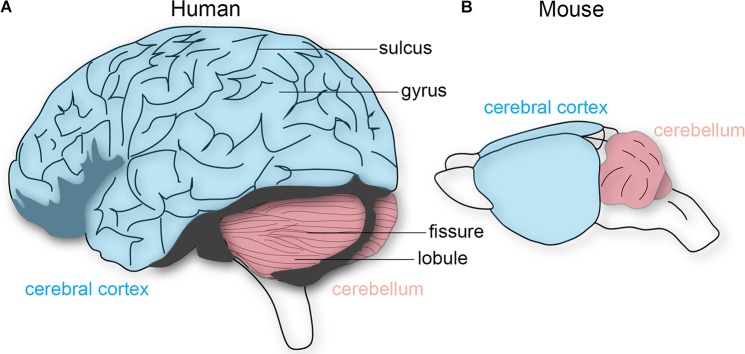
Schematic depiction of the human and mouse brain. **(A)** The human cerebral cortex (blue) and cerebellum (pink) both have characteristic folding patterns. The cerebral cortex contains gyri (peaks) that are interrupted by sulci (valleys). An equivalent architecture is observed in the cerebellum, which is comprised of lobules and fissures. **(B)** The mouse cerebellum is folded whereas the cerebral cortex is not. The brains are not drawn to scale.

How should we determine whether structural features of the brain constitute functionally separate areas or nuclei, without under-dividing or over-dividing? Kaas (1982) proposed five criteria to test for this problem: that distinct cortical areas and nuclei should have differences in cytoarchitecture, a relatively complete single representation of the sensory surface, unique inputs and outputs, different response properties, and dissociable behavioral impairments after lesions. In the cerebellum, the repeating Purkinje cell circuit is subdivided into olivocorticonuclear modules, which also contain micromodules that could either represent distinct or combinations of functional entities (Apps et al., 2018). For instance, lesions made into distinct olivary sub-nuclei, key components of the cerebellar modules, result in specific behavioral deficits during movement (Horn et al., 2010). Moreover, *in vivo* recordings in mice and rats demonstrate different Purkinje cell firing properties that are dependent on location within the cerebellar cortical patterns (Xiao et al., 2014; Zhou et al., 2014). However, a more fundamental feature that has been a useful and reliable landmark for locating distinct areas is the overlying brain morphology. Most notably, the folds overlying the cerebral cortex and cerebellum. The relationship between external morphology and functional organization has also been essential for studying the brain functions of extinct species, as we are only able to examine skull endocasts. The cerebral cortex contains a series of gyri that are separated by sulci, whereas the cerebellum has lobules separated by fissures (**Figure 1**). Cortical folds are consistent across individuals (and between some mammalian species), whereas cerebellar folds are highly conserved as the major pattern across species, from birds through mammals (Larsell, 1970). In primates, larger brains preserve neuronal packing density and generally have more and deeper folds (Herculano-Houzel et al., 2007), which are especially evident in the massive and proportional expansion of the human neocortex and cerebellar cortex. There has been a long-standing debate as to how folding is accomplished, and how it might relate to brain function (Mota and Herculano-Houzel, 2015). We next discuss and debate some of the theories and experimental evidence.

### Mechanics of Folding a Neural Sheet

In recent years, much focus has been placed on testing the tension theory proposed by Van Essen (1997). The general tenet of his theory is that cortico-cortical axons physically pull on regions of the cortex and result in gyri formation, and almost by default, the sulci form as well. Other prominent theories, such as the radial gradient hypothesis, propose that an increase in the expansion of supragranular cortical layers relative to the infra-granular layers causes buckling of adjacent regions (Richman et al., 1975). In the differential tangential expansion hypothesis, it is proposed that tangential expansion of the cortex causes an increase in tangential pressure, and as a consequence buckling acts to reduce the pressure (Le Gros Clark, 1945; Ronan et al., 2013). In this case, the theory leans on Brodmann’s findings on cytoarchitectural differences, which allow for pattern-specific folding. Following this theory, the stability of folding patterns reflects the stability of expansion forces in a given region. It is worth mentioning that these theories share the idea that cortical folding upon itself is necessary for packing the large brain into the small skull, an idea that could be challenged by experimental evidence almost 70 years ago (Barron, 1950).

Moreover, empirical studies of the cerebral cortex argue against features of these theories, and an emerging counter argument is that the timing of developmental events simply could not facilitate tension or buckling (Ronan and Fletcher, 2015). We could make similar arguments for the cerebellum. Van Essen (1997) posits that tension along parallel fibers, the axons of granule cells, could explain why the cerebellar cortex is highly elongated but also folded like an accordion into lobules. Arguing against Van Essen’s tension-based mechanism is the fact that cerebellar cortical folding starts embryonically whereas most parallel fibers form postnatally, and, despite the number of fiber tracts entering, no one class could drive the folding because afferents enter the cerebellum starting from mid-embryogenesis and continue to sequentially invade the structure throughout postnatal development (White and Sillitoe, 2013). Moreover, the several theories above do not take into account the highly dynamic nature of circuit formation as fibers are also pruned away, and the target cells—not the axons—are key regulators of wiring processes that should also impact tension and buckling based on these theories (Uesaka et al., 2014). One attractive idea, based on the original hypothesis of Altman and Bayer (1997), is that the shape of folds is driven by the precise timing of the appearance of specialized “anchoring centers” at the future base of each cerebellar fissure and the subsequent coordinated proliferation and migration of granule cells down Bergmann glia astrocytes (Sudarov and Joyner, 2007). But, the fact that cerebellar folds are well conserved and reliably patterned in every animal points to genetic mechanisms that support what might be a functional necessity. Indeed, sonic hedgehog (SHH) morphogen signaling and engrailed transcription factor function, among other pathways (Ryan et al., 2017), could play integrated roles in shaping the cerebellum (Sudarov and Joyner, 2007; Blaess et al., 2008).

The idea of neuronal proliferation driving folding extends to the cerebral cortex as well (Hevner, 2006; Ronan and Fletcher, 2015; Sun and Hevner, 2016). However, it is not just the progenitors that exhibit spatial organization. It was postulated that perhaps folds encompass units with functional restriction (Welker, 1990). Genetic clonal analysis uncovered a modular mode of development for cerebellar granule cells, with lineages restricted within folds (Legue et al., 2015). In the cerebral cortex, neurons born from a common lineage form local clusters that migrate into distinct cortical, hippocampal, and striatal regions (Sultan et al., 2016); although this idea is challenged by Mayer et al. (2016). These early patterns are reminiscent of protomaps (Rakic et al., 2009). More importantly, developmental clusters produce the maps required for adult function and behavior. In the cerebellum, clusters might not directly determine function, but we know they are part of a framework for establishing topography, which is important for function. There is also a possibility that clusters, and the genes that are differentially expressed within each subset, instruct the cellular and molecular properties that eventually produce the diversity of cerebellar-related behaviors.

Still, there are many features of the theories put forth by Van Essen et al. (2018) that could beautifully explain a number of processes required for cerebral cortical and cerebellar folding, especially from the view of evolution. It is likely that these and other ideas (Na(V), Smith et al., 2018; extracellular matrix, Long et al., 2018) will merge to unveil the full explanation for how neural tissue accomplishes its folding (Meng et al., 2018). In addition, perhaps it is time to consider new model systems (Matsumoto et al., 2017). Recent work used human microcephaly as a starting point for understanding cortical size and complexity. Knowing that mutations in the abnormal spindle-like microcephaly-associated (*Aspm*) gene are common in the human disease but have surprisingly little impact on mouse cortex, Johnson and colleagues developed an *Aspm* knockout ferret (Johnson et al., 2018). The rationale for choosing this model was the more complicated gryrification of the cerebral cortex compared to rodents and the greater diversity of neuronal progenitors. In the *Aspm* model, the approach proved fruitful as they uncovered essential roles for radial glia in controlling cortical expansion. It would be interesting to examine the role of *Aspm* in the ferret cerebellum, considering the milder, but evident cerebellar defects (Johnson et al., 2018).

### Topography Relates to Function

**FIGURE 2:**
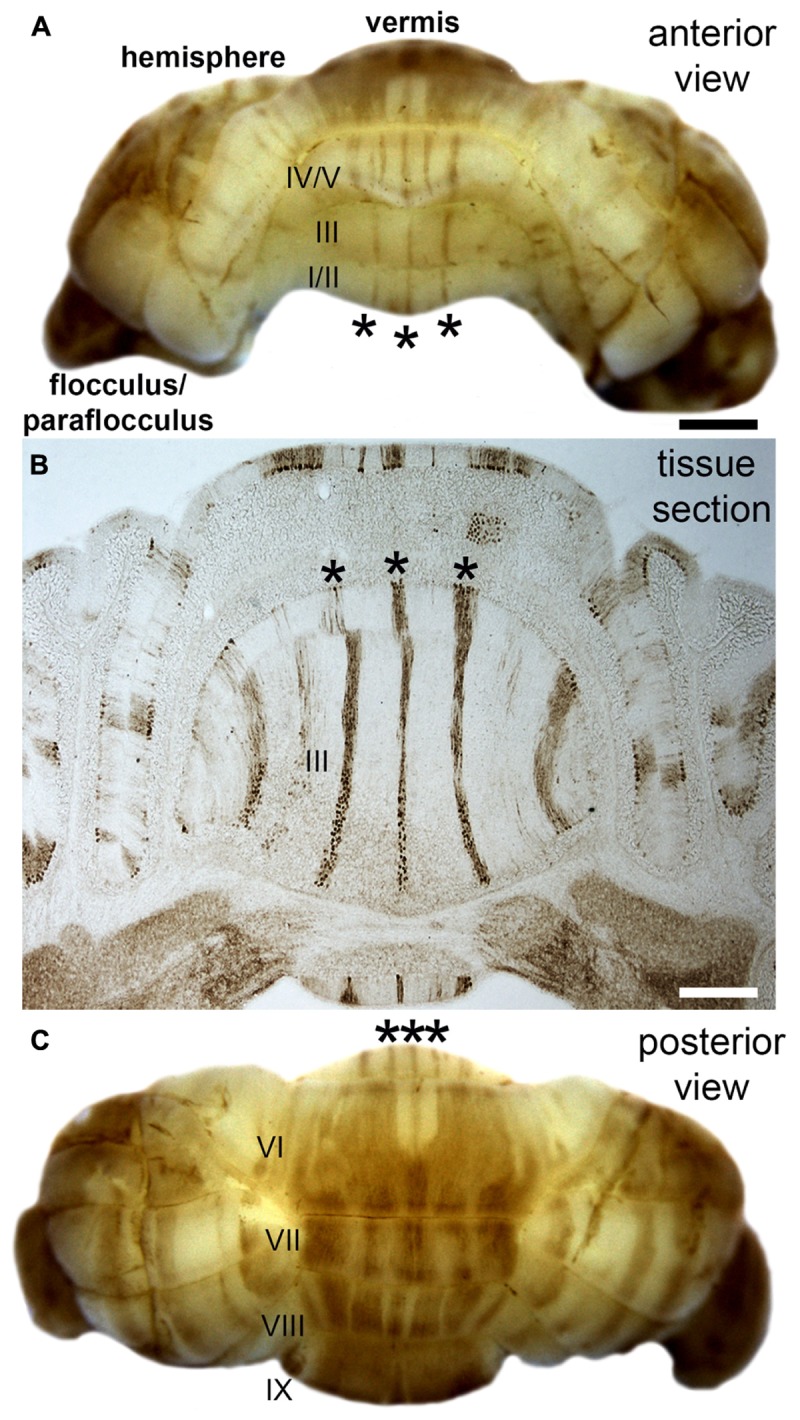
Purkinje cell patterning in the adult mouse cerebellum. **(A)** Anterior view of the mouse cerebellum stained in wholemount with a monoclonal antibody to zebrin II/aldolaseC. **(B)** Coronal tissue section cut through the anterior cerebellum illustrating the pattern of zebrin II stripes in the anterior lobules (asterisks in all panels). Although the intervening stripes are not immunoreactive for zebrin II, they do express other markers that define this complementary set of stripes. **(C)** Posterior view of the mouse cerebellum stained in wholemount with zebrin II. The lobules in the vermis are labeled with Roman numerals according to the nomenclature of Larsell (Larsell, 1970). Scale bar in **A** = 1 mm (applies to **C**), and the scale bar in **B** = 500 μm. The images were modified with permission from White et al. (2012).

Folds roughly correlate to functional domains. In the cerebral cortex, the four principle lobes each have a predominant set of functions. Different sensory, motor, and cognitive behaviors are mapped to distinct regions (a famous correlate was the removal of the hippocampi in patient HM and the resulting specific inability to form new memories), and in some cases, each with specific cytoarchitectures. A remarkable evolutionary adaptation reflecting the structure-function relationship is seen in star-nosed moles, which have 22 fleshy foraging appendages that ring their nostrils. Each appendage is represented in the somatosensory cortex, where they form a “cortical star” that mimics the structure (Catania, 2012). An equivalent topographical mapping of sensory representations onto the cortical surface is revealed in ocular dominance columns in the visual cortex (Hubel and Wiesel, 1979), barrels in the somatosensory cortex (Li and Crair, 2011), patch matrix compartments in the striatum (Bloem et al., 2017), and odorant maps in the olfactory system (Bozza et al., 2002). One of the best-studied examples of brain organization is the Purkinje cell zonal map (**Figure 2**; Miterko et al., 2018). The topographic inputs and outputs of Purkinje cell zones form functional modules (Ruigrok, 2011). Gene expression reveals patterns of stripes that demarcate the modules (cerebellar stripe patterns are obvious in Purkinje cells; **Figure 2**). Modules are derived from lineage patterning mechanisms that instruct specific classes of sensory afferents to target particular regions of the cerebellum (and to some extent particular folds). Modules are therefore comprised of neurons, glia, and terminals that are wired together with a specific topography. It is proposed that modules are organized into patterns that provide an efficient packaging framework for circuits to encode behaviors and that could allow parallel processing of information during complex behaviors (Horn et al., 2010). From a developmental perspective, one could ask what are the embryonic origins of two adjacent circuits that perform complementary functions during a given behavior? For instance, the hindlimb and forelimb cooperate during locomotion, and it is not a coincidence that limb sensory axons are placed side by side in the module map (Gebre et al., 2012). The wiring of the cerebellar map is genetically determined (Sillitoe et al., 2010) and sculpted to precision by activity (White et al., 2014), and the same is true for retinotopic maps in the visual cortex (Cang et al., 2008). The topographic structure of the input and output pathways as well as their internal maps (e.g., Purkinje cell patterns) is therefore intimately linked to circuit function and the behaviors that the circuits support.

### Taking Shape in Space and Time

Besides folds, brain and region size is another important feature established during development that is thought to influence global and local function. As folds became increasingly more complex, and potentially co-evolving with the necessity for higher order brain functions, the need for larger mammalian brains possibly arose. Accompanying this increase in brain size is an increase in the number of neurons and glia, and an increase in the diversity within each cell class. More specifically, it is thought that the increase in the number of astrocytes is to preserve its roles of synaptogenesis and regulating metabolic demand in a more complex and robust neural environment (Nedergaard et al., 2003; Oberheim et al., 2006).

Individual brain regions also enlarged as a result of adaptation. For example, the neocortex in primates exponentially increased in size (Finlay and Darlington, 1995) and the hippocampus of foraging birds enlarged as the need for improved social interaction and spatial memory mounted (Sherry et al., 1992). Epigenetic events during development, such as loss of a sensory organ (Kahn and Krubitzer, 2002), have been shown to influence the size and function of cortical areas, though within limits established by genetic programs. The observation that magnification has accompanied the acquisition of complex behaviors raises the question of how regions expand during development, and with this process again comes the obligatory issue of folding. Genetic programs during development create balance between proliferation and cell death, and when genes such as *LIS1*, *DCX*, *ASPM*, *EMX2*, *FGF*, and *Gpr56* affect the normal processes of neuron and glia formation and migration, a host of conditions including lissencephaly and polymicrogyria ensue (Ronan and Fletcher, 2015). Interestingly, defective SHH signaling, one of the most powerful morphogens in mammalian development and an essential component of cerebellar folding during late embryogenesis and early postnatal development in mice (Corrales, 2006), also controls cerebral cortical growth and folding by modulating cell proliferation (Wang L. et al., 2016; Wang Y. et al., 2016). It should therefore not be surprising that the outcome of conditions with defective SHH signaling can be extremely severe, as is the case when the catalytic subunit of phosphoinositide 3-kinase (*PIK3CA*) is mutated causing brain and body overgrowth. In the brain, *PIK3CA*-induced overgrowth causes bilateral dysplastic megalencephaly, hemimegalencephaly, and focal cortical dysplasia, the most common cause of intractable pediatric epilepsy (Roy et al., 2015).

Clear behavioral symptoms also result from the reversed problem, i.e., when reduced growth is triggered, as in the case of *reeler* mutations, the result is a small brain with no folds. In this scenario, the organized layering of the cerebral cortex, hippocampus, and cerebellum is also disrupted (Sudarov et al., 2011; Guy and Staiger, 2017). A recent epidemic with similar consequences is Zika virus infection, which causes microcephaly with cognitive disability. The Zika virus impairs Musashi-1 function in neural progenitors, leading to decreased neurogenesis in both the cortex and the dentate gyrus and therefore an overall decrease in size (Chavali et al., 2017; Christian et al., 2018). Moreover, the Zika virus disorganizes the granule cell layer and strata, in addition to predisposes infants to brain calcifications (Christian et al., 2018). Accompanying these structural deficits to the brain are insults to neural circuit development. The Zika virus promotes gliosis, impairs myelination, disrupts synaptogenesis, and alters the levels of SHH and FGF (Christian et al., 2018; Thawani et al., 2018). But, during normal development it should be noted that even before secreted molecules such as SHH, FGF, and REELIN impact cell placement, lineage history and birthdate initiate a spatial and temporal order of cellular invasion of a territory (Sudarov et al., 2011). In addition, in the cerebral cortex, arealization maps the areas of invasion by a transcription factor-mediated process (O’Leary et al., 2007) that can also involve repurposed axon guidance cues in rodents and primates (Homman-Ludiye and Bourne, 2014).

Axon guidance itself plays a multitude of roles in shaping structures for function. For instance, functional systems are determined by how effective pioneer tracts are in setting up circuits, how guidepost cells mediate the proper trajectory of axons, and how chemoaffinity cues such EPH-EPHRIN and cadherin signaling establish circuit maps through axon-target recognition. Interestingly, progenitors not only drive proliferation for regional expansion, but recent evidence indicates that they also produce the NETRIN guidance cue for proper long-range targeting of axons (Dominici et al., 2017; Varadarajan et al., 2017). Remarkably, yet an additional role for SHH is to mediate axon-axon guidance to shape structure into the optic chiasm crossing (Peng et al., 2018), a necessary step in forming the precise retinotectal topography that underlies vision.

It is interesting to speculate that changes in these molecular processes and axon routing could account for the subtle but significant variations in the sizes of brain regions observed in different strains of inbred mice (Hikishima et al., 2017). To solve how this occurs, dissecting the impact of a large number of intersecting genetic pathways *in vivo* would be impractical. However, one could consider highlighting the relationship between structure and function, even within the context of folding, in a disease-relevant model using organoids. The cellular, molecular, and structural features of Zika, and the more basic mechanisms of brain folding, were rapidly worked out with organoids (Cugola et al., 2016; Garcez et al., 2016; Qian et al., 2017; Karzbrun et al., 2018). Perhaps human organoids could also be helpful in developmental conditions in which the structural defects are unclear yet function is perturbed. One example is hydrocephalus. Hydrocephalus is a severe developmental disorder characterized by an excessive build-up of CSF, which causes pressure on the surrounding tissue. In rare cases, the hydrocephalus may be spontaneously corrected such that brain scans no longer show the ventricular enlargement (Dreazen et al., 1989). This is surprising because hydrocephalus alters hindbrain shape, and if shape is no longer defective, then endogenous repair mechanisms could be at play. This is intriguing because recent work shows that a specific progenitor population in the cerebellum can rescue granule cells post-injury (Wojcinski et al., 2017).

More complicated are conditions such as autism with overt behavioral deficits and covert structural deficits. While a reduction in gray matter volume has been found in the cerebellum, hippocampus, amygdala, and parietal lobe of children with autism spectrum disorder (ASD), others have reported an overall increase in brain volume as a hallmark feature of ASD (Carlisi et al., 2017; Riddle et al., 2017). Studies on the underlying genes associated with ASD pathogenesis suggest that a range of functions are disrupted such as synaptic maintenance and motor control (Antoine et al., 2018; Golden et al., 2018; Tatavarty et al., 2018). However, inconsistent structural findings and the seemingly endless number or combination of genetic causes have complicated the process of studying function in autism and other diseases. As a result, the development of new therapeutic approaches has been modest. Recent methodological advances could hold promise.

Organoids and iPSC approaches could be tailor-made to address the mechanism of diseases with either structural or circuit-based defects. In their remarkable study, Orly Reiner and colleagues were inspired by physics and tackled the problem of cortical folding using organoids. They used “wrinkling” as a proxy for folding and uncovered that the process is driven by mechanical instability (Karzbrun et al., 2018). Furthermore, they showed in their organoid system that a *LIS1*^+/−^ mutation reduced wrinkling, and at the cellular level, the defects arose through a combination of defects in nuclear motion, extracellular matrix, and cytoskeleton. A major question to answer—and model—is whether circuit function could be fully restored if the structure of the developing brain is rescued in time. In addressing this question, it could be that the regional differences in stem cell and progenitor population number and capabilities have to be considered. For instance, radial glia-like cells in the outer subventricular zone could promote cortical folding complexity (Fietz et al., 2010; Hansen et al., 2010; Wang et al., 2011). It would be interesting if the inner versus outer granule cell progenitors in the external granular layer have differential effects on cerebellar lobulation. Moreover, there may be a great number of genes, such as *ARHGAP11A* (which encodes a Rho guanosine triphosphatase-activating protein), that control the species-specific complexity of folding (Florio et al., 2015), not only in cortex but other regions as well. To disentangle whether function could be rescued if folding is corrected, the timing of when the activity of such progenitors or genes is initiated is critical.

### Structure-Function Reciprocity

Evidently, injury to the brain and more specifically to regions such as the cortex and cerebellum has severe consequences on function (Dahlem et al., 2016). In addition to the direct consequences of missing an area, one has to consider that compensation for loss of function, even in long-range circuits such as the brain to spinal cord connectivity or cerebro-cerebellar connectivity, adds considerable complexity to the structure-function relationship (Hollis et al., 2016). In stroke, the functional improvements are dependent on structural plasticity that induces changes at the synapse (Cooperrider et al., 2014). Indeed, in the cerebellum, topographic molecular mechanisms determine spatial patterns of synaptic plasticity (Wadiche and Jahr, 2005). Synaptic connectivity also follow an architectural heterogeneity of the targets. For example, despite the textbook view of a canonical Purkinje cell, these cells vary in their form and function depending on where in the cerebellum they are located (Cerminara et al., 2015). Purkinje cells have different dendritic structures depending on where they are in a fold. And, at the action potential level, subsets of Purkinje cells have very unique functional (firing) properties that align almost perfectly with the modular stripe topography of the cerebellar cortex (Xiao et al., 2014; Zhou et al., 2014).

Similarly, one can imagine that that cellular diversity and the dense connectivity plan between cerebral cortical cells should impact computations (Jiang et al., 2015), especially when synaptic structural variations, such as size and neurotransmitter phenotype, are taken into account (Larramendi et al., 1967). We can also no longer ignore that glial diversity will shape synaptic output (John Lin et al., 2017). In the cerebellum, loss of AMPA receptors in Bergmann glia results in motor coordination defects (Saab et al., 2012). Bergmann glia are patterned according to Purkinje cell stripes (Reeber et al., 2018) and are critical for foliation (Ma et al., 2012; Li et al., 2014). It would therefore be interesting to consider whether the three-dimensional plan of the cerebellum with its Cartesian coordinate-like map (Sillitoe and Joyner, 2007) uses Bergmann glia heterogeneity and their influence over neuronal activity to help sculpt the folding and shape of the cerebellum. Could the anchor centers at the base of each fold be defined by particular stripe and zone features? And, are these features unique to each fold? Perhaps the glia-neuron heterogeneity plays a role in differentiating the vermis from hemisphere folds, which require additional curvature even seen in the mouse cerebellum. Certainly, in addition to the differences in functional roles of the vermis compared to the hemispheres, there exists developmental (Govek et al., 2018) as well as disease related distinctions (Tan et al., 2018) that drive shape. It is intriguing to speculate that the initiation and growth of the additional folia and sub-folia in higher order mammals could be facilitated by cellular and molecular heterogeneity in the highly conserved cerebellar map (Sillitoe et al., 2005; Marzban and Hawkes, 2011), and perhaps other classes of glia such as oligodendrocytes and NG2+ cells could in theory promote specific aspects of folding over time. Might there be equivalent features of heterogeneity in the cerebral cortex and hippocampus to support their folding? There is certainly enough heterogeneity for this to be possible.

### Behavioral Implications

Considering the above findings together, we are reminded that cellular and structural heterogeneity, even in structures with as high a regional diversity as the cerebral cortex, map to a fundamental functional plan (Mountcastle, 1957). But there is a problem raised by this heterogeneity in that how do these different circuits work together to ultimately drive behavior? This problem is in part solved by neuronal synchrony. Whether it is in the cerebral cortex, cerebellum, or another area, circuits that are linked by cell lineage or connectivity can be synchronously activated in a manner that provides flexibility in controlling behavior (Welsh et al., 2002). But, does long-range connectivity between brain areas with different canonical circuit structures allow for different types of plasticity to work together to influence behavior (Caligore et al., 2017)? Such a model would provide one mechanism for increasing functional flexibility.

**FIGURE 3:**
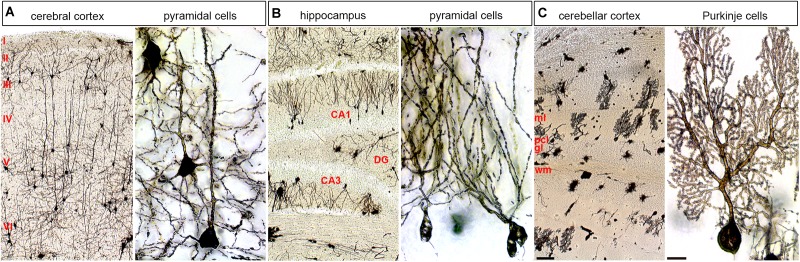
Cellular diversity and specificity in different layered brain structures. The adult mouse brain was stained using a modified Golgi–Cox method. **(A)** Cerebral cortex shown at low and high power. **(B)** Hippocampus shown at low and high power shown at low and high power. **(C)** Cerebellum shown at low and high power shown at low and high power. Pyramidal cells are shown at high power for the cerebral cortex and hippocampus and Purkinje cells are shown for the cerebellum. ml, molecular layer; pcl, Purkinje cell layer; gl, granular layer; wm, white matter; DG, dentate gyrus. The six main layers of the cerebral cortex are labeled with Roman numerals. The scale bar in **C** (cerebellar cortex) = 100 μm and applies to all three low power images. The scale bar in **C** (Purkinje cells) = 20 μm and applies to all three high power images.

Also, localized cellular diversity in each cortical column or cerebellar module could add computational diversity (**Figure 3**). These discrete functional features are built into the brain, but some degree of structure has to be in place first. Though, it is intriguing that blocking neuronal function in the embryonic cerebellum causes lifelong changes in circuit function and behavior without changing structure (White and Sillitoe, 2017). This is consistent with findings that the wiring of topographic circuits–a proxy for arising function–is not strictly linked to the morphology of overlying folds (Sillitoe et al., 2008). Still, the intricate folding (cerebral cortex and cerebellum) and layering (cerebral cortex, cerebellum, hippocampus, and other regions; **Figure 3**) in the different regions of the brain are necessary developmental steps in establishing circuits.

## Summary

Regional differences in proliferation set up the structural flexibility that folds and shapes the brain. Genetic cascades control the process, but they also initiate the patterned wiring of circuits. Establishment of structure-function relationships is therefore dependent on topographic circuits, which define the connectivity patterns of networks of neurons with specific physiological properties, which jointly control particular behavioral outcomes. Cues such as SHH play multiple roles throughout development, tying several aspects of brain growth, patterning, wiring, and behavior together. But there is a still a major gap in our understanding of how the precise structure formed in one region relates to that in connected regions. For simplicity, we have discussed somewhat independently the cerebral cortex and cerebellum as models of two areas that are structurally vastly different but are massively interconnected (Bostan et al., 2013) with widely overlapping functional involvements. Moreover, conditions such as ASDs could involve long-range functional interactions from the cerebellum to cortex (Stoodley et al., 2017). Could coordinated inter-regional growth perform critical functions, with common spatial and temporal developmental features? And how would this get processed to impact behavior? The synchronous neuronal activity within structurally defined local circuits could be integrated with circuits of similar design in the connected regions. Proper network structures allow neural computations to actively harness the power of “communication through coherence” as a means of integrating multiple brain systems (Fries, 2015). Indeed, there is growing evidence that this may be how the cerebellum communicates with the hippocampus and the cerebral cortex during non-motor function (McAfee et al., 2017). To further resolve this, future studies could exploit the phenotypes of rare human diseases (Wangler et al., 2017) as a means to systematically define how developmental programs drive the coordinated assembly of structure and function in the brain.

## Ethics Statement

Animal studies were performed under an approved Institutional Animal Care and Use Committee (IACUC) protocol at Baylor College of Medicine.

## Author Contributions

LM and RS wrote the first draft. LM, EL, DH, and RS edited the manuscript and finalized the paper.

## Conflict of Interest Statement

The authors declare that the research was conducted in the absence of any commercial or financial relationships that could be construed as a potential conflict of interest.
